# Quadrivalent meningococcal tetanus toxoid-conjugate booster vaccination in children aged 10–12 years: phase III randomized trial complementary analysis of immune persistence 3–6 years after priming

**DOI:** 10.1038/s41390-024-03760-w

**Published:** 2024-12-13

**Authors:** James Peterson, Katherine Galarza, Siham Bchir, Celine Zocchetti, Isabelle Bertrand-Gerentes, Betzana Zambrano

**Affiliations:** 1 J. Lewis Research, Salt Lake City, UT USA; 2 Scientific & Medical Affairs, Sanofi, Swiftwater, PA USA; 3 Global Biostatistical Sciences, Sanofi, Marcy l’Étoile, France; 4 Global Medical Affairs, Sanofi Lyon, France; 5 Global Clinical Development Strategy, Sanofi, Montevideo, Uruguay

## Abstract

- Immune persistence following primary vaccination with a single dose of meningococcal quadrivalent conjugate vaccines, MenACYW-TT/MCV4-CRM, at age 10–12 years was demonstrated.
- Most participants primed with MenACYW-TT and MCV4-CRM maintained seroprotective titers against all serogroups, suggesting continued protection.
- Priming with MenACYW-TT resulted in higher persistent titers for serogroups C, W, and Y than MCV4-CRM.
- A MenACYW-TT booster induced a robust immune response; almost all participants achieved seroprotection against all serogroups.
- This analysis supports the ACIP recommendations for routine administration of a primary dose of a meningococcal ACWY conjugate vaccine at 11–12 years, with a booster dose at 16 years.

A phase IIIb study (MET59; NCT04084769) was conducted in adolescents and young adults in the USA to evaluate the persistence of meningococcal antibodies 3–6 years after priming with either MenACYW-TT (MenQuadfi®; Sanofi, Swiftwater, USA) or MCV4-CRM (Menveo, GSK) conjugate vaccines, and to describe the safety profile and immune response following administration of a booster dose of MenACYW-TT.^1^ A total of 570 adolescents and young adults, aged ≥13 to <26 years, who had received a single dose of MenACYW-TT or MCV4-CRM 3–6 years earlier during studies MET50 (NCT02199691)^2^ and MET43 (NCT02842853),^3^ were enrolled in study MET59.^1^

Given that protection has been shown to wane, a booster dose can provide continued protection during adolescence since this is an age group at high risk of meningococcal disease.^4^ This complementary analysis of MET59 data was conducted to focus on a specific subset of adolescents whose vaccination timing aligned closely with the Advisory Committee on Immunization Practices (ACIP) recommended schedule, which advises a priming dose between the ages of 11 and 12 years, followed by a booster dose 3–6 years later, to evaluate the persistence of the immune response after priming with either MenACYW-TT and MCV4-CRM.^4^

Study MET59 was a phase IIIb, open-label, partially randomized, parallel group, active-controlled, multi-center study conducted at 29 centers in the USA and one center in Puerto Rico. A detailed description of the MET59 study design has been previously published.^1^ A total of 570 participants with a mean age (standard deviation [SD]) of 15.4 (±1.4) years (range: 13–24 years) were enrolled. For this complementary analysis, data from participants aged 10–12 years of age on the day of priming with MenACYW-TT or MCV4-CRM in studies MET50 and MET43 were analyzed.

Immune persistence of a single priming dose of MenACYW-TT or MCV4-CRM was assessed by serum bactericidal antibody assay using human complement (hSBA). Vaccine seroprotection rates (hSBA titers ≥1:8) and hSBA geometric mean titers (GMTs) were assessed 30 days post-priming with MenACYW-TT or MCV4-CRM in studies MET50 and MET43, and at Day 0 of study MET59 (3–6 years after priming), prior to MenACYW-TT booster administration.

The MenACYW-TT booster immune response was assessed as follows: hSBA GMTs at Day 0 and Day 30 after a booster vaccination according to the priming vaccine (MenACYW-TT or MCV4-CRM); seroprotection rates (hSBA titers ≥1:8) at Day 0 and Day 30 in MenACYW-TT primed participants (MenACYW-TT alone, MenACYW-TT+MenB-T, and MenACYW-TT+4CMenB) and MCV4-CRM-primed participants.

Overall, 470 of the 570 participants (82.5%) included in study MET59 were 10–12 years of age on the day of priming with MenACYW-TT (*n* = 340) or MCV4-CRM (*n* = 130). At the time of enrollment in study MET59, the mean age (SD) of participants primed with MenACYW-TT was 15.1 (±1.01) years and 15.6 (±0.81) years for those primed with MCV4-CRM. The majority of participants were Caucasian (MenACYW-TT primed, 87.4%; MCV4-CRM primed, 91.5%).

The persistence of the immune response was evaluated at Day 0 of MET59, prior to administration of the MenACYW-TT booster. Across the four serogroups, seroprotection rates at Day 30 post-priming with MenACYW-TT were 95.3% for serogroup A, 98.8% for serogroup C, 100.0% for serogroup W, and 97.9% for serogroup Y; by 3–6 years, these declined to 71.1% for serogroup A, 86.8% for serogroup C, 88.2% for serogroup W, and 82.3% for serogroup Y (Table 1). Seroprotection rates at Day 30 post-priming with MCV4-CRM were 80.6% for serogroup A, 73.2% for serogroup C, 93.5% for serogroup Y, and 88.7% for serogroup W; by 3–6 years, these declined to 73.1% for serogroup A, 48.5% for serogroup C, 75.4% for serogroup Y, and 51.5% for serogroup W (Table 1). GMTs also declined in both primed groups but remained higher than those measured prior to priming vaccination (Fig. 1), indicative of long-term persistence of immune response.

**Table 1:** Persistence (hSBA seroprotection rates) of MenACYW-TT and MCV4-CRM immunity 3–6 years after priming and immunogenicity of MenACYW-TT booster in participants 10–12 years of age (per protocol analysis set 2).

Serogroup	MenACYW-TT primed: seroprotection rate, hSBA titers ≥ 1:8 (95% CI)			MCV4-CRM primed: seroprotection rate, hSBA titers ≥ 1:8 (95% CI)		
	Persistence		Booster	Persistence		Booster
	D30 – Post-priming dose *N* = 340	D0 – Pre-booster dose *N* = 340	D30 – Post booster dose *N* = 340	D30 – Post-priming dose *N* = 130	D0 – Pre-booster dose *N* = 130	D30 – Post-booster dose *N* = 130
**A**	95.3 (92.5, 97.3)	71.1 (65.9, 75.9)	99.4 (97.9, 99.9)	80.6 (72.6, 87.2)	73.1 (64.6, 80.5)	99.2 (95.8, 100.0)
**C**	98.8 (97.0, 99.7)	86.8 (82.7, 90.2)	100.0 (98.9, 100.0)	73.2 (64.4, 80.8)	48.5 (39.6, 57.4)	100.0 (97.2, 100.0)
**W**	100.0 (98.9, 100.0)	88.2 (84.3, 91.4)	100.0 (98.9, 100.0)	93.5 (87.7, 97.2)	75.4 (67.1, 82.5)	100.0 (97.2, 100.0)
**Y**	97.9 (95.8, 99.2)	82.3 (77.8, 86.2)	100.0 (98.9, 100.0)	88.7 (81.8, 93.7)	51.5 (42.6, 60.4)	100.0 (97.2, 100.0)

Per protocol analysis set 2 comprised participants from the full analysis set who provided samples at Day 30 with no protocol deviation.

*CI* confidence interval, *D* day, *hSBA* serum bactericidal antibody assay using human complement.

**Fig. 1:**
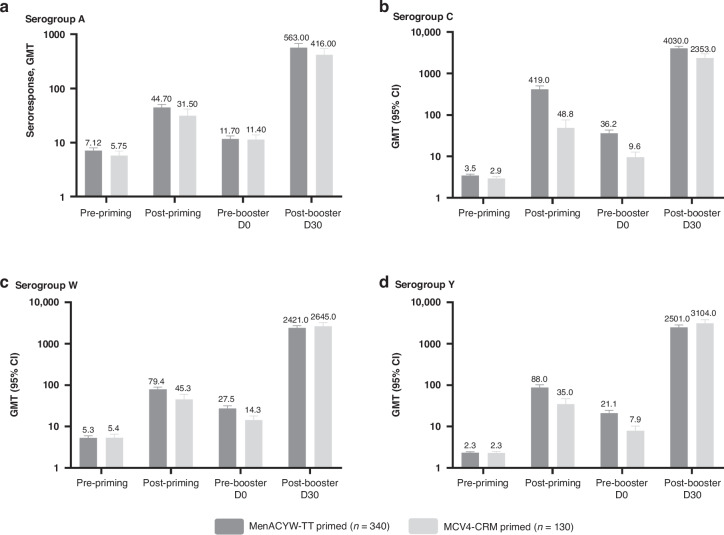
hSBA GMTs before and after primary dose of either MenACYW-TT or MCV4-CRM, and before and after MenACYW-TT booster. Persistence of hSBA GMTs to serogroup A (**a**), serogroup C (**b**), serogroup W (**c**) and serogroup Y (**d**) following priming vaccination with MenACYW-TT or MCV4-CRM. MenACYW-TT primed (pooled groups 1, 3 & 4) consists of participants who were MenACYW-TT primed in the previous studies (MET50 or MET43); MenACYW-CRM primed (Group 2) consists of participants who were MCV4-CRM primed in the previous study (MET50). The pre-priming dose GMTs were measured on D0 of studies MET50 and MET43; the post-priming dose GMTs were measured 30 days after administration of the priming vaccine in studies MET43 and MET50. Error bars display the 95% confidence intervals. CI confidence interval, D day, GMT geometric mean titer, hSBA serum bactericidal antibody assay using human complement, n total number of participants.

At Day 30 after administration of MenACYW-TT booster, nearly all participants in both the MenACYW-TT- and MCV4-CRM-primed groups achieved seroprotective titers (hSBA titer ≥1:8) for each serogroup (Table 1). An anamnestic response was observed post-booster with MenACYW-TT, with increases in GMTs much higher than those observed after priming vaccination across all serogroups (Fig. 1).

This analysis shows that priming vaccination with a single dose of MenACYW-TT at 10–12 years of age induces an antibody immune response against all four vaccine serogroups that persists for at least 3–6 years. Additionally, at least 70% of participants primed with MenACYW-TT maintained seroprotective titers (hSBA titers ≥1:8), suggesting continued protection against invasive meningococcal disease in this population up to the ACIP-recommended age to receive a booster dose (at approximately 16 years of age).^4^ Of note, a priming dose of MenACYW-TT appears to consistently result in persistence of a higher level of hSBA antibody for serogroups C, W, and Y, as assessed by seroprotection (hSBA titers ≥1:8) rates and GMTs, compared with a priming dose of MCV4-CRM. Persistence was similar in both MenACYW-TT-primed and MCV4-CRM-primed groups for serogroup A. Booster vaccination with MenACYW-TT induced robust hSBA antibody responses against all vaccine serogroups with almost all participants achieving seroprotection (hSBA titers ≥1:8) 30 days post-booster in both MenACYW-TT- and MCV4-CRM-primed groups.

In conclusion, the results of our analysis support the ACIP-recommended schedule for routine administration of a priming dose of meningococcal ACWY conjugate vaccine at 11–12 years of age followed by a booster dose at 16 years of age.^4^

The datasets generated and/or analyzed during the current study, including the raw data, are not publicly available in order to safeguard the privacy of participants and the confidentiality and protection of their data, as well as protect commercially sensitive information. Qualified researchers may request access to participant level data and related study documents including the clinical study report, study protocol with any amendments, blank case report form, statistical analysis plan, and dataset specifications. Participant level data will be anonymized and study documents will be redacted to protect the privacy of our trial participants. Further details on Sanofi’s data sharing criteria, including required permissions to access the data, eligible studies, and process for requesting access can be found at: https://www.vivli.org/.
